# Cry1Ba1-mediated toxicity of transgenic *Bergera koenigii* and *Citrus sinensis* to the Asian citrus psyllid *Diaphorina citri*

**DOI:** 10.3389/finsc.2023.1125987

**Published:** 2023-04-24

**Authors:** Vladimir Orbović, Seyed Ali Ravanfar, Diann S. Achor, Turksen Shilts, Freddy Ibanez-Carrasco, Rahul Banerjee, Choaa El-Mohtar, Lukasz L. Stelinski, Bryony C. Bonning

**Affiliations:** ^1^ Citrus Research and Education Center, Institute of Food and Agricultural Sciences (IFAS), University of Florida, Lake Alfred, FL, United States; ^2^ Entomology and Nematology Department, Institute of Food and Agricultural Sciences (IFAS), University of Florida, Gainesville, FL, United States

**Keywords:** genetic transformation, pesticidal protein, *Bacillus thuringiensis*, Bt toxin, insect resistant transgenic plants

## Abstract

The Asian citrus psyllid, *Diaphorina citri*, vectors the bacterial causative agent of citrus greening disease, which has severely impacted citrus production on a global scale. As the current repeated application of chemical insecticides is unsustainable for management of this insect and subsequent protection of groves, we investigated the potential use of the bacteria-derived pesticidal protein, Cry1Ba1, when delivered *via* transgenic citrus plants. Having demonstrated transformation of the Indian curry leaf tree, *Bergera koenigii*, for Cry1Ba1 expression for use as a trap plant, we produced transgenic plants of Duncan grapefruit, *Citrus paridisi*, Valencia sweet orange, *Citrus sinensis*, and Carrizo citrange, *C. sinensis x Poncirus trifoliata*, for expression of Cry1Ba1. The presence of the *cry1ba1* gene, and *cry1ba1* transcription were confirmed. Western blot detection of Cry1Ba1 was confirmed in most cases. When compared to those from wild-type plants, leaf discs from transgenic Duncan and Valencia expressing Cry1Ba1 exhibited a “delayed senescence” phenotype, similar to observations made for transgenic *B. koenigii*. In bioassays, significant reductions in the survival of adult psyllids were noted on transgenic *B. koenigii* and Valencia sweet orange plants expressing Cry1Ba1, but not on transgenic Duncan grapefruit or Carrizo citrange. In contrast to psyllids fed on wild type plants, the gut epithelium of psyllids fed on transgenic plants was damaged, consistent with the mode of action of Cry1Ba1. These results indicate that the transgenic expression of a bacterial pesticidal protein in *B. koenigii* and Valencia sweet orange offers a viable option for management of *D. citri*, that may contribute to solutions that counter citrus greening disease.

## 1 Introduction

The Asian citrus psyllid (ACP), *Diaphorina citri* (L.) Kuwayama is the vector of the plant pathogenic proteobacterial species ‘*Candidatus* Liberibacter asiaticus’ (CLas) and ‘*Candidatus* Liberibacter americanus’ (CLam), the presumed causative agents of citrus greening (also known as huanglongbing) in Asia and the Americas (1, 2). Trees infected with these bacteria have high mortality and dramatic decreases in fruit yield with dire consequences in Florida and other citrus growing regions (3–5). Management of ACP populations has been a primary strategy against the spread of citrus greening into groves with newly planted citrus trees. However, the cost of citrus grove management significantly increased with the repeated application of chemical insecticides, which also selected for insecticide resistance in ACP (6, 7). Environmental issues associated with the potential impacts of insecticides on non-target organisms is an additional area of concern (8), that warrants consideration of alternative strategies for psyllid management in citrus.

The successful use of transgenic crops engineered to produce bacteria-derived pesticidal proteins for suppression of pestiferous insects has been demonstrated in multiple cropping systems, including cotton, maize and forest trees such as poplar (9–13). These structurally diverse bacterial proteins are derived from *Bacillus thuringiensis* (Bt) (14). In citrus, such pesticidal proteins could be deployed *via* transgenic fruit-yielding citrus, or *via* transgenic trap plants, i.e. an alternative host plant that is highly attractive to the psyllid but is not a host for CLas or CLam. As psyllids move into citrus groves from neighboring smaller habitats (15), trap plants surrounding the grove would function to intercept ACP prior to grove entry. We and others have previously reported on *B. thuringiensis*-derived pesticidal proteins with toxicity against ACP (16, 17), and the production of transgenic Indian curry leaf plant, *Bergera koenigii* expressing one of the psyllid-active proteins, Cry1Ba1 (18). Transgenic *B. koenigii* could be used as a trap plant when planted in or around citrus groves to attract psyllids away from fruit-yielding citrus. In this scenario, psyllids that feed on the trap plant would die from ingestion of Cry1Ba1 before reaching the citrus grove. Transgenic citrus trees or trap plants that suppress ACP populations would benefit citrus production in conjunction with other management tools through reducing the need for frequent insecticide applications thereby decreasing both management costs and potential negative impacts on the environment. Transgenic citrus or trap plants may also contribute to reducing the spread of citrus greening when used in conjunction with other strategies that target CLas or CLam.

Following transformation of *B. koenigii* for potential use as a trap plant for psyllid management (18), our goal for this project was first to produce transgenic plants of three different citrus cultivars, Duncan grapefruit, Valencia sweet orange, and Carrizo citrange, expressing the *cry1ba1* gene. Having confirmed the presence of the *cry1ba1 *gene, transcription, and translation, we then sought to determine the impact of transgenic plants for which protein expression was confirmed on the physiology, behavior and survival of ACP. Based on the results of this study, the potential for use of transgenic citrus plants and alternative approaches for their deployment for ACP management are discussed.

## 2 Materials and methods

### 2.1 Vector construct and plant transformation

Construction of the pCAMBIA2301-derived binary vector pBa1 for expression of Cry1Ba1 has been described previously (18). This vector includes sequences encoding kanamycin resistance (neomycin phosphotransferase II; *nptII*), green fluorescent protein (GFP) and the citrus codon-optimized active core of Cry1Ba1 along with a GNA precursor leader sequence (19). Cry1Ba1 expression is driven by the constitutive CaMV 35S promoter for expression in all tissues including phloem, the site of psyllid feeding (20, 21). The pBa1 vector was mobilized into *Agrobacterium tumefaciens* EHA105 competent cells. Previously described protocols (18, 22) were employed for the genetic transformation of Duncan grapefruit (*Citrus paradisi* Macf.), Valencia sweet orange [*Citrus sinensis* (L.) Osbeck], and Carrizo citrange [*Citrus sinensis* Washington Navel sweet orange X *Poncirus trifoliata* (L.) Raf.]. In brief, etiolated seedlings of these cultivars were cut into 10-15 mm long explants and incubated in freshly prepared suspension of *A. tumefaciens*. After co-incubation with *Agrobacterium*, explants were placed on co-cultivation medium, CCM (23), for two days and moved to regeneration medium, RM, for shoot induction and elimination of *Agrobacterium*. After five weeks, shoots that sprouted from seedling explants were inspected for fluorescence of GFP under a fluorescence stereomicroscope (Leica MZ16 FA; Leica, Bannockburn, IL) with a 480 nm excitation blue light. Shoots that exhibited GFP fluorescence were micro-grafted on Carrizo rootstock plants. The plantlets made of grafted shoots and rootstocks were transferred to cubic pots (5 cm^3^) covered with inverted magenta boxes to maintain high humidity and left on a light bench with constant white light (fluence rate = 50 μmol/m^2^s). After two months in the laboratory, these plantlets were moved to the greenhouse, where they were acclimated to grow in larger pots.

### 2.2 Confirmation of Cry1Ba1 presence and transcription by quantitative real-time PCR analysis

Quantitative PCR (qPCR) was used to assess the presence of *cry1ba1* in the plant genome (24). Sample preparation and qPCR were conducted as described previously (18). qPCR was performed using three different primer pairs targeting different regions of *cry1ba1* (18) in a 7500 Thermal Cycler (Applied Biosystems, Waltham, MA, USA) using Power SYBR™ Green RNA-to-C_T_1 step kit (Applied Biosystems Catalog # 4391178) without the RT-step. The quality of DNA isolated from transgenic and control plants was confirmed by amplification of the *actin* gene (18). The qPCR reaction volume of 10 µl was comprised of 5 µl 2XSYBR mix, 0.5 µl 10 mM forward primer, 0.5 µl 10 mM reverse primer, 0.1 µl SYBR enzyme and 1 µl template DNA diluted 1 to 50 to negate the effect of PCR inhibitors present in the resuspended DNA. The program was run at 95°C for 10 min, 40 cycles of 95°C for 15 sec, and 60°C for 1 min. A melting curve was run at the end of the qPCR program.

The transgenic plants generated were first screened by RT-qPCR for the production of *cry1ba1* transcripts. Total RNA was extracted from leaves of transgenic or wild type (WT) citrus plants with the RNeasy Plant Mini Kit (Qiagen, Germantown, MD). cDNA was synthesized from 1μg of RNA using the iScript cDNA Synthesis kit (Bio-Rad Labs, Hercules, CA). ACT primers were used to amplify an *actin* fragment as a reference for normalization of transcripts, and the c*ry1ba1* target site was amplified using primers Ba1(f) 5’-GCCTATTCACGGAGTTCCAA-3’ (nucleotides 1122 to 1142 within the c*ry1ba1* sequence) and Ba1(r) 5’-CAACTGAAGACCTGGTGATTC-3’ (nucleotides 1216 to 1235) (18). Appropriate amplification of *cry1ba1* with these primers was confirmed prior to their use in these assays. The qPCR validations were carried out in duplicate using the iTaq Universal SYBR Green mix (Bio-Rad Labs) in an Eco Real-Time thermocycler (Illumina) with the following program: 95°C for 2 min, 42 cycles at 95°C for 5 s, 57°C for 10 s and 72°C for 15 s. The specificity of PCR amplification was verified by melt curve analysis (from 55°C to 95°C). *cry1ba1* transcript levels in transgenic plants relative to WT were calculated using the 2^−ΔΔCt^ method (25).

### 2.3 Assessment of Cry1Ba1 expression by western blot

Having confirmed *cry1ba1* transcription by transgenic plants, western blot analysis was conducted on tissues from each plant to test whether Cry1Ba1 was produced. For production of Cry1Ba1 protein for use as a positive control in western blots, the pMH19 plasmid expressing Cry1Ba1 [bases 1 to 2037 (26)] was transformed into *Escherichia coli* XL-1. The positive colonies were grown on Terrific Broth (TB)-medium containing ampicillin (100 μg/ml) and 2% glucose at 37°C for 3 days. The cells were harvested and the pellet suspended in lysis buffer (50 mM Tris-HCl pH8, 5 mM EDTA, 100 mM NaCl, 8 μl 50 mM PMSF/gram pellet). The pellet was further washed 2–3 times with buffer (20 mM TrisHCl, pH 7.5, 1 M NaCl, 1% Triton X-100) and crystals were solubilized in 50 mM NaHCO3 20 mM NaCl, 10 mM DTT pH10. The solubilized Cry1Ba1 was activated by treating with bovine trypsin (Sigma, St. Louis, MO) at a 1:10 ratio of trypsin: protein in buffer at pH 7.5, with incubation at 37°C for 3 h. Trypsin was removed by incubating the sample with benzamidine sepharose (GE health care, Chicago, IL) at RT for 30 min by shaking slowly and collecting the clear supernatant by centrifuging at 4,000 rpm at RT for 10 min. Total protein in the solubilized fractions was estimated using Bradford’s Reagent. Cry1Ba1 was separated on 4–20% gradient polyacrylamide gels (Bio-Rad) and transferred to the PVDF membrane using standard procedures. IgG purification from polyclonal anti-Cry1Ba1 raised in rabbits (PRF&L, Canadensis, PA, USA) was conducted as described previously (27). To assess the sensitivity of the purified IgG fraction, 0.02 µg to 2 µg of activated Cry1Ba1, were transferred to a PVDF membrane (Amersham International PLC, Amersham, UK). The membrane was blocked with 1X TBS 0.2% Tween 20 and 5% BSA. Cry1Ba was detected with the purified Cry1Ba IgG (dilution 1:250) and an HRP-coupled secondary antibody (Thermo Fisher Scientific, Waltham, MA, USA: dilution 1:5,000) followed by a Pierce chemiluminescent substrate (Thermo Fisher Scientific).

For western blot analysis, the soluble protein fraction was prepared from plant tissues as follows: 100 mg of leaf tissues was pulverized into powder with liquid nitrogen in a mortar and pestle. The powdered tissue was then solubilized overnight in 500 μL of extraction buffer (0.1% IGEPAL, 50 mM Tris-HCl pH 7.5, 10 mM MgCl_2_ and 150 mM NaCl) supplemented with 1X protease inhibitor (cOmplete™, Mini, EDTA-free Protease Inhibitor Cocktail: Roche, Basel, Switzerland) at 4°C. The solution was centrifuged at 16,100 g for 15 minutes at 4°C with soluble proteins remaining in the aqueous supernatant layer. Protein concentrations for solubilized leaf tissue samples were determined by Bradford assay (28).

Solubilized proteins from WT and transgenic citrus plants were separated in 4–20% SDS PAGE gels (15 or 20 µg protein per lane). Proteins from the gels were transferred to a PVDF membrane (Amersham). The blocking and detection of Cry1Ba1 was as described above for assessment of purified IgG sensitivity. Following analysis for detection of Cry1Ba1, the membrane was stripped with a mild stripping buffer (15 g glycine, 1 g SDS and 10 ml Tween-20 pH 2.2 for 1 L) and re-probed with anti-GFP antibody (Invitrogen anti-GFP polyclonal; A-11122, Thermo Fisher; dilution 1:5000) and anti-rabbit HRP secondary antibody (dilution 1:5000).

The production of transgenic *Bergera koenigii* and confirmation of *cry1ba1* transcription were as described by Ravanfar et al. (18).

### 2.4 Impact of Cry1Ba1 expression on plant phenotype

Four Valencia and four Duncan plants for which *cry1ba1* transcription and translation had been confirmed, were used to determine whether the “delayed senescence” phenotype described for transgenic *B. koenigii* expressing Cry1Ba1 (18) would be observed. For each genotype, transgenic plants were paired with four WT controls of the same genotype, size, and age. A single detached leaf per plant was used to create 15 mm diameter leaf discs cut with a cork hole borer. Discs were placed into Petri dishes (35 mm; Fisher brand, Thermo Fisher Scientific, Waltham, MA), one disc per dish containing a 1.5% agar bed. The agar in the dishes maintained moisture (and leaf turgor) for approximately 2 weeks. The phenotype of the leaf discs taken from each of four plants from each of four treatments was recorded on day 3, day 7 and every 7 days thereafter until 60 d after leaf harvest and scored as 1-green, succulent; 2- green, dry; or 3- brown, wilted, dead or dying in appearance. Due to the destructive nature of this approach, a single replicate of four plants per citrus variety was conducted for observation of the “delayed senescence” phenotype.

### 2.5 Impact of transgenic citrus on Asian citrus psyllid

Asian citrus psyllids, *Diaphorina citri*, from an insecticide susceptible and CLas/CLam-free culture maintained in a greenhouse at the Citrus Research and Education Center, University of Florida, Lake Alfred, FL were used in bioassays to assess the survival of adult ACP on test transgenic Indian curry leaf and citrus plants. The insects were reared on various host plants (described above) for at least two generations prior to conducting biological evaluations on those same hosts to control for possible effects of host adaptation. Rearing occurred at 27–28°C, with 60–65% relative humidity, and a 14:10 h (light: dark) photoperiod.

Transgenic *B. koenigii*, Duncan grapefruit, as well as Carrizo citrange and Valencia orange plants (T0 generation) at ~50 cm in height for which transcription of *cry1ba1* had been confirmed were used for bioassays. These were *B. koenigii* 2, 6, 16a (18); Valencia 15, 16, and 22; Duncan 3, 5, and 23; and Carrizo 2, 4, 7, 8, 10, and 14. Prior to initiation of bioassays, plants leaves were washed twice per week using a 2% Dawn dish detergent spray solution (Procter and Gamble, Cincinnati, OH). Twenty to 30 minutes after detergent was applied, plants were rinsed with clean water. Subsequently, plants were repositioned into individual cages and maintained at 23 ± 3°C, 60% RH, and a 16:8 h (Light: Dark) photoperiod with a maximum photosynthetic radiation of 215 µmol s^−1^ m^−2^. Plants were watered twice per week and fertilized twice per month with an alternating schedule of a 24-8-16 NPK solution at 4 g L^−1^ (Miracle-Gro All Purpose Plant Food; Scotts Miracle-Gro Products, Marysville, OH) and a 6-4-6 (N–P–K) granular fertilizer at 1 g per pot (Expert gardener Gro Tec. Inc. Madison, GA).

After withholding food from *D. citri* for 4 hours, 10 adults of mixed sex and age were released into bags sealed tightly around stems to prevent insect escape. Mortality was observed every other day for 8 days. For all Duncan and Valencia plants, flush, feather-like structures were apparent in both Cry1Ba1 transgenic and control plants. Plant bioassays were replicated independently three times for transgenic *cry1ba1* Valencia, Duncan, and Indian curry leaf transgenic plants alongside wild type plants and six times with trangenic Carrizo plants and respective controls.

The settling preference of adult *D. citri* between transgenic Duncan grapefruit plants with *cry1ba1* versus control plants was evaluated with an open-air choice assay. Control plants were of the same variety and approximate age and size. Newly emerged adult psyllids were collected and host preference was assessed using a choice assay as described elsewhere (29) with slight modifications. Briefly, plants were inserted into a 1.8×1.8×1.0 m cage custom made from 0.5 cm thick, white, and opaque acrylic. Plants were positioned at opposite sides of the cage 1 m apart. Position of treatment and control plants was randomized prior to each replicate. Afterward, 25 psyllids were vacuum‐suctioned into separate 50-mL vials. Subsequently, collection vials were introduced into the center of the cage containing two plants (a plant with *cry1ba1* to one side and the control plant to the other side). The cap was removed from the collection vial allowing *D. citri* to move freely. The experiment was conducted under light conditions generated by a fluorescent 900-lux light bulb under temperature-controlled conditions of 27 ± 1°C, 63 ± 2% RH, and under a L14∶D10 h photoperiod. Each run of the host preference experiment contained three independent replicates with different treatment and control plants that were conducted on different days. The entire experiment was repeated twice. Psyllid preference was determined 24 hours after release by counting all psyllids alighting on each plant. Psyllids were considered settled when found in a feeding position with their abdomen at a 45° to the plant surface. Approximately 75% of all released psyllids were found on either of the two plants presented for settling choice. Assays were conducted with two control plants that determined no positional bias within the assay cage as evidenced by a 50:50 psyllid settling response between two control plants of similar size and age.

### 2.6 Transmission electron microscopy of ACP fed on transgenic and control plants

To look for potential physiological damage associated with Cry1Ba1-mediated toxicity, transmission electron microscopy (TEM) was used to examine the gut epithelium of ACP fed on Cry1Ba1 transformed Valencia, Duncan grapefruit and *B. koenigii* (Indian curry leaf), or control plants. The plants used here were the same as those used for toxicity bioassays described above, with a total of three plants each for Valencia, Duncan, *B. koenigii* or control (wild type). Adult psyllids (20 per plant) were maintained on the plants for 8 days. Adult ACP were collected for TEM analysis with >40 live insects pooled for each treatment. The head and tip of the abdomen was removed from each psyllid, and the torsos fixed for 16 h at 4°C in 1X PBS buffer containing 3% (v/v) glutaraldehyde. Samples were then postfixed for 4 h at room temperature in 2% (v/v) osmium tetroxide, dehydrated in acetone, and embedded in Spurr’s Resin (30). Cross sections were examined by light microscopy for location of the midgut and blocks sectioned from the anterior or posterior ends of 8 to 10 psyllids per treatment. Ultrathin sections (90 nm) mounted on copper grids were stained with 2% (w/v) aqueous uranyl acetate and lead citrate (31) and observed using an FEI Morgagni 268 Transmission Electron Microscope (FEI Company, Hillsboro, OR, USA).

### 2.7 Statistical analysis

Data collected for psyllid bioassays comparing psyllid survival on transgenic plants with *cry1ba1* and control plants were analyzed using a Kaplan-Meier survival analysis (32), and a Log-Rank test for pairwise comparisons using the RStudio environment version 3.6.3 (33). Behavioral response by ACP between the two host outcomes was analyzed using chi-square contingency tables also in R (33).

## 3 Results

### 3.1 Citrus transformation and confirmation of *cry1ba1* insertion

The citrus cultivars Valencia sweet orange, Duncan grapefruit and Carrizo citrange were transformed with *cry1ba1*, *nptii* and *gfp*. Antibiotic resistance was used as the basis for selection of transformants and green fluorescence used to visualize heterologous protein expression in tissues of the transformants and to determine transformation rates. For transformation of Duncan grapefruit we used 12,832 explants that were co-incubated with *Agrobacterium*. The number of shoots and buds that sprouted from Duncan explants was 17,598, for which GFP fluorescence was only detected in 71 shoots resulting in a transformation success rate of 0.40% (**Table 1**). Of these, 49 shoots were chimeric and 22 were fully transformed. To produce transgenic Valencia plants, 10,016 explants were co-incubated with *Agrobacterium* and 6,242 shoots that sprouted from those explants were examined for GFP fluorescence. Fluorescence was detected in 17 shoots comprised of seven that were fully transformed and 10 that were chimeric. The transformation rate for Valencia was 0.27%. Compared to Valencia and Duncan, the transformation rate for Carrizo citrange was the highest at 0.69%. For Carrizo, 1,920 explants were co-incubated with *Agrobacterium*, and 2,023 shoots sprouted from those explants. Of these, 14 were transgenic with GFP fluorescence. Three of the 14 shoots were chimeric and 11 fully transformed. A total of seven Carrizo citrange plants were produced and survived the transition to soil. In all cases, only fully transformed shoots were grafted on rootstock plants. Some of the grafted plants grew poorly and some did not survive the transition from *in vitro* culture to soil. A total of 27 transgenic plants of different citrus cultivars expressing *cry1ba1* gene were produced: Fourteen Duncan grapefruit cultivar plants, six Valencia sweet orange and seven Carrizo citrange. These plants acclimated well to grow in pots. The presence of the transgene in the plants was assessed by use of qPCR with three sets of *cry1ba1* primers (**Table 2**). The 1:1:1 ratio of qPCR Ct values amplified using three different sets of primers from the same isolated DNA indicates that the full *cry1ba1* gene was present as part of the T-DNA.

**Table 1:** Transformation rate for citrus cultivars used in this study.

Cultivar	Number of shoots and buds inspected	GFP positive	Transformation rate (%)
Duncan	17598	71	0.40
Valencia	6242	17	0.27
Carrizo	2023	14	0.69

**Table 2:** Confirmation of *cry1ba1* presence in representative transgenic plants.

	Primer set-1		Primer set-2		Primer set-3		ACT	
Plant	Ct	Tm	Ct	Tm	Ct	Tm	Ct	Tm
H_2_O	U	NA	U	NA	U	NA	U	N/A
VAL WT	U	NA	U	NA	U	NA	20.69 ± 0.39	77.44 ± 0.00
**VAL 15**	23.78 ± 0.56	74.00 ± 0.11	24.11 ± 0.13	74.74 ± 0.11	24.14 ± 0.33	75.11 ± 0.11	23.96 ± 0.31	77.21 ± 0.19
**VAL 16**	24.18 ± 0.18	74.28 ± 0.00	24.31 ± .0.20	74.90 ± 0.11	24.56 ± 0.15	74.90 ± 0.11	23.52 ± 0.24	77.01 ± 0.28
**VAL 22**	23.57 ± 0.13	74.12 ± 0.11	23.68 ± 0.19	74.92 ± 0.11	23.82 ± 0.07	75.11 ± 0.11	24.76 ± 0.06	77.15 ± 0.11
DUN WT	U	NA	U	NA	U	NA	20.26 ± 0.10	77.46 ± 0.11
**DUN 0**	19.58 ± 0.03	73.70 ± 0.18	19.51 ± 0.10	74.55 ± 0.11	19.63 ± 0.08	74.74 ± 0.11	20.60 ± 0.09	76.20 ± 0.11
**DUN 20**	25.05 ± 0.09	74.10 ± 0.11	25.34 ± 0.47	74.91 ± 0.11	25.17 ± 0.09	74.97 ± 0.00	21.40 ± 0.10	77.14 ± 0.11
CAR WT	U	NA	U	NA	U	NA	17.15 ± 0.22	77.01 ± 0.00
**CAR 2**	21.82 ± 0.14	73.85 ± 0.32	21.91 ± 0.01	74.23 ± 0.00	21.12 ± 0.29	74.47 ± 0.11	19.32 ± 0.19	76.33 ± 0.21
**CAR 4**	19.49 ± 0.00	73.98 ± 0.11	19.44 ± 0.26	74.78 ± 0.00	19.48 ± 0.12	74.78 ± 0.00	18.41 ± 0.10	76.70 ± 0.11
**CAR 7**	20.51 ± 0.21	73.98 ± 0.11	20.10 ± 0.23	74.60 ± 0.19	20.30 ± 0.22	74.72 ± 0.11	19.19 ± 0.58	76.57 ± 0.43
**CAR 14**	23.68 ± 0.21	73.67 ± 0.00	23.57 ± 0.27	74.47 ± 0.28	23.79 ± 0.29	74.66 ± 0.11	19.87 ± 0.57	76.57 ± 0.28
**CAR 21**	18.24 ± 0.01	73.39 ± 0.39	17.88 ± 0.14	74.16 ± 0.28	18.21 ± 0.11	74.35 ± 0.28	17.96 ± 0.20	76.45 ± 0.19

VAL, Valencia sweet orange; DUN, Duncan grapefruit; CAR, Carrizo citrange; ACT, actin; Ct, cycle threshold; Tm, primer melting temperature; U, undetermined; NA, not applicable.

Quantitative PCR analysis with three sets of primers (18) confirming the presence of *cry1ba1* in transgenic plants. Data for control plants (WT) and the qPCR negative control (H_2_O) are also shown.

### 3.2 Confirmation of *cry1ba1* transcription and translation

Transcription of *cry1ba1* was confirmed for all transgenic plants with variable levels of transcript accumulation detected (**Table 3**). Using the purified Cry1Ba1 IgG that was sufficiently sensitive for detection of 0.02 µg Cry1Ba1 (i.e. a detection limit of 100 ppm in western blots based on loading 20 µg plant sample per lane; **Figure S1**), translation of the *cry1ba1* gene in transgenic plants was confirmed in most instances (**Table 3**; **Figure S1**). The goal of the western blots was to confirm Cry1Ba1 production and not to compare relative protein levels between lines. For some samples, although a protein band of the size expected for the toxic protein (~ 50kDa) was not observed, a strong immunoreactive band of lower molecular mass was apparent. These plants were scored as “negative” (**Table 3**). Cry1Ba1 detection was not consistent with multiple blots required to confirm expression in some cases (**Table S1**). Western blots for detection of GFP that were used as controls for the western blotting procedure, confirmed GFP expression by all transgenic Duncan, Valencia, and Carrizo plants (**Figure S1**).

**Table 3:** Confirmation of transcription and translation for plants transformed for expression of Cry1Ba1.

Plant	Transcript Levels	Western blot detection	
		Cry1Ba1	GFP
DUN WT	0.0	**-**	**-**
Dun 1	2.9	+	+
Dun 3	176.2	+	+
Dun 5	197.3	+	+
Dun 6	24.6	+	+
Dun 9	819.5	+	+
Dun 11	561.4	-*	+
Dun 12	580.2	+	+
Dun 13	120.6	+	+
Dun 18	51.7	+	+
Dun 20	106.3	+	+
Dun 23	161.5	-*	+
Dun 25	65.5	+	+
VAL WT	0.0	**-**	**-**
Val 15	56.6	+	+
Val 16	77.6	+	+
Val 17	144.4	+	+
Val 19	346.1	+	+
Val 22	806.4	+	+
VAL N	NT	-*	+
CAR WT	0.0	-	-
Car 2	317	+	+
Car 4	304	+	+
Car 7	2100	+	+
Car 8	833	+	+
Car 10	1736	+	+
Car 14	292	+	+
Car 21	150	+	+

Transcript levels of *cry1ba1* in transgenic plants compared to wild type calculated with reference to *actin* using the 2^−ΔΔCt^ method. Western blots conducted for both Cry1Ba1 and GFP detection are shown in **Figure S1**, with a summary table provided (**Table S1**). -, not detected; +, protein of the expected size detected; * immunoreactive bands of less than the expected size detected.

### 3.3 Impact of Cry1Ba1 production on transgenic plants and ACP plant preference

**Figure 1:**
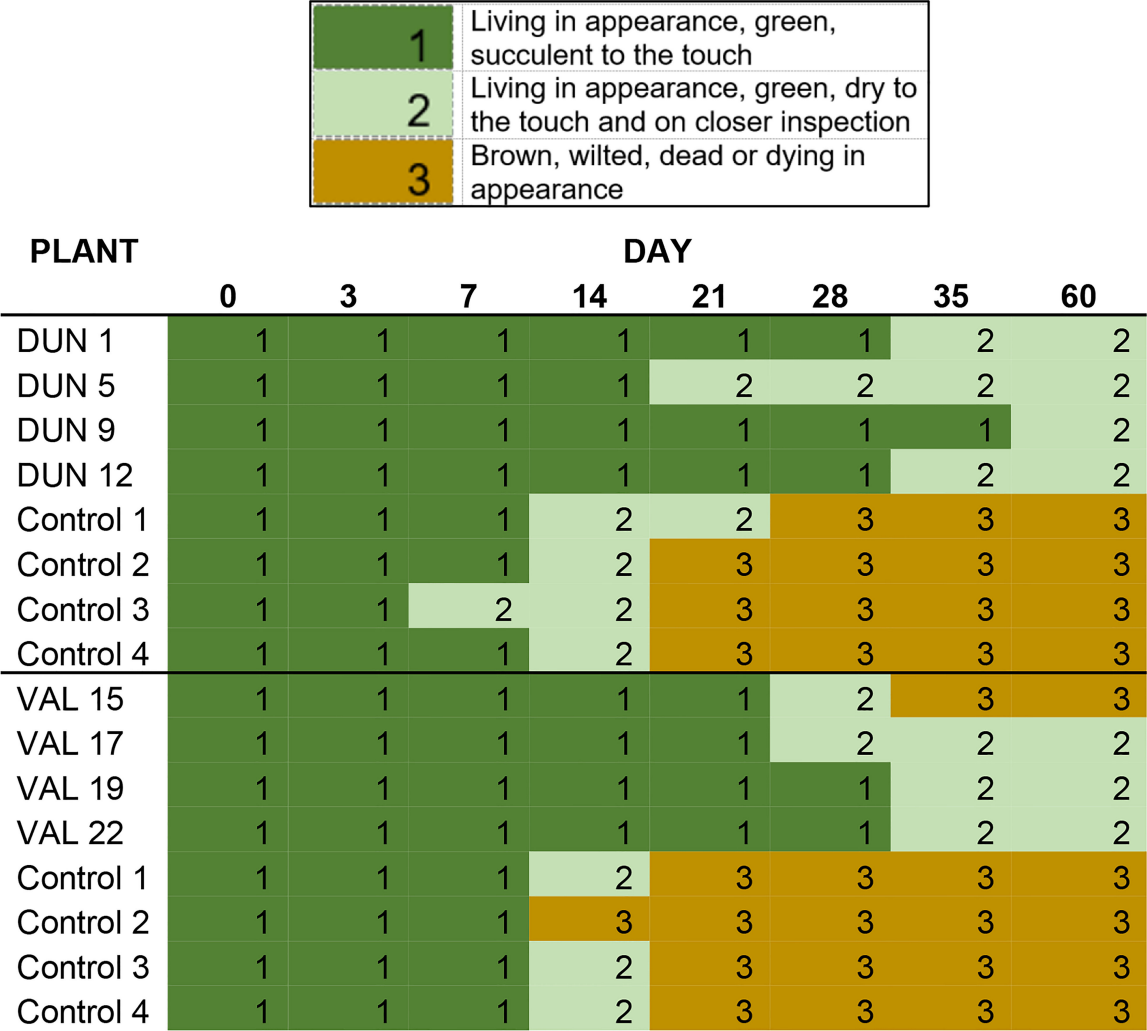
“Delayed senescence” phenotype of Cry1Ba1 transgenic plants. Graphic shows the delayed desiccation and browning of leaf discs maintained on agar plates from Cry1Ba1-expressing Duncan or Valencia compared to control plants. In contrast to those from transgenic plants, discs from the control plants had dried and mostly turned brown by day 21.

Leaf discs taken from transgenic Valencia and Duncan plants expressing Cry1Ba1 exhibited a reduced tendency to dry out and turn brown in appearance relative to control plants. While discs from control plants had all dried and turned brown by day 21 (**Figure 1**), those from only one transgenic Valencia plant turned brown by day 35. Discs from the other seven transgenic plants (Valencia and Duncan) remained green and living in appearance but dry to the touch until the end of the experiment (day 60). To assess whether psyllids would avoid Cry1Ba1-expressing transgenic plants, behavioral assays were conducted. Adult psyllids exhibited no preference (χ^2 ^= 1.07, P = 0.459) in choice test experiments, settling with equal frequency on transgenic Duncan or control plants (**Figure S2**).

### 3.4 Impact of transgenic plants on ACP survival

**Figure 2:**
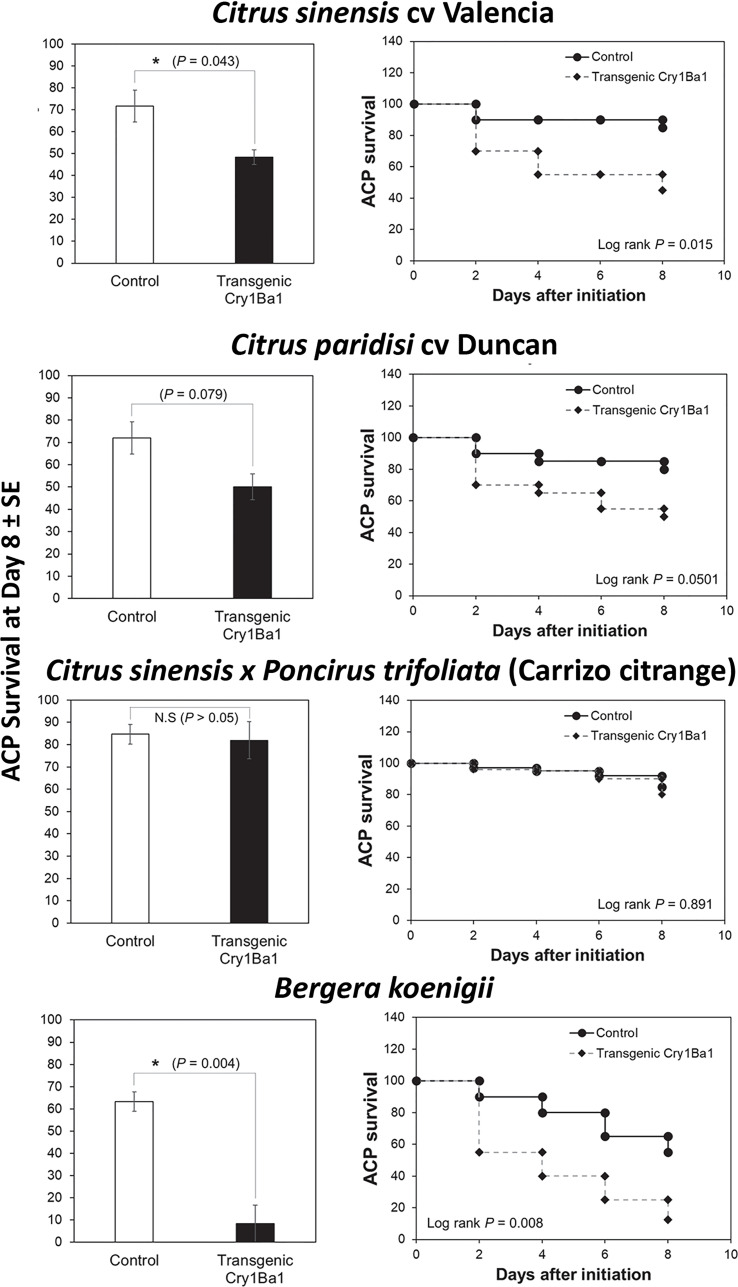
Reduced survival of Asian citrus psyllids on transgenic *B. koenigii* and *C. sinensis* expressing Cry1Ba1. Bars represent the mean Asian citrus psyllid (ACP) survival ± standard error (SE), with statistical differences indicated (*, significantly different; N.S, not significantly different; Log-Rank test for pairwise comparisons). ACP survival during the course of the bioassay is also shown for one of three independent replicates per treatment. Curves depict the Kaplan-Meier survival probabilities of ACP adults after access to transgenic plants.

Bioassays using whole plants showed that survival of ACP adults was significantly reduced on Indian curry leaf plant and Valencia sweet orange expressing Cry1Ba1 relative to control plants without transformation (**Figure 2**). Although a drop in ACP survival on transgenic Duncan by day 8 was noted, it was not statistically significant from survival on control plants (p = 0.079). No impact on psyllid survival was seen for transgenic Carrizo compared to control plants (p > 0.05).

**Figure 3:**
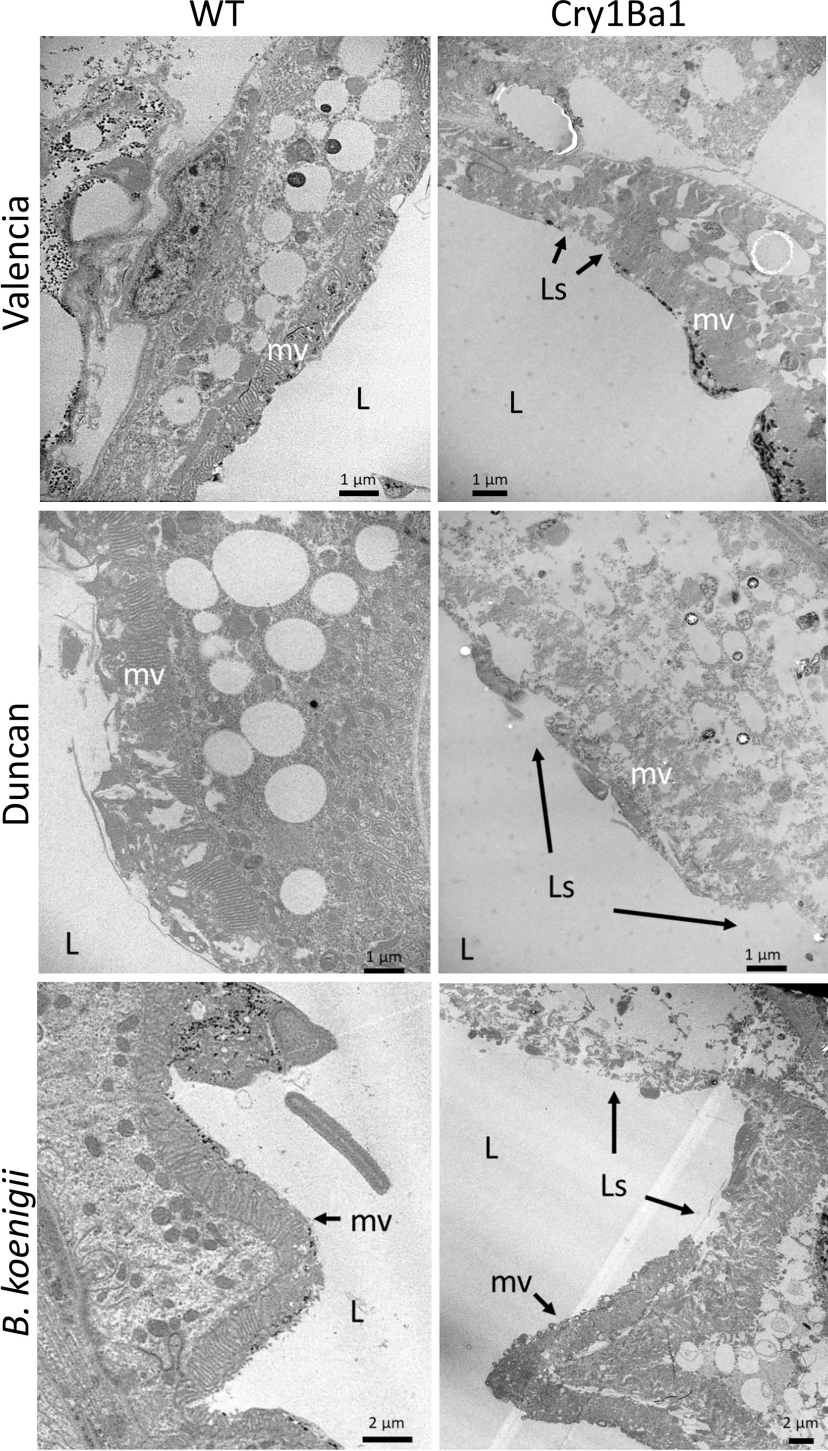
Damage to gut epithelial tissues of Asian citrus psyllid consistent with Cry1Ba1-mediated toxicity. Representative transmission electron micrographs showing the gut epithelia of ACP fed on WT or transgenic Cry1Ba1-expressing plants. While the microvilli lining the gut epithelium of ACP fed on WT plants are intact, the microvilli of ACP fed on transgenic plants were severely disrupted with multiple lesions apparent. L, gut lumen; mv, microvilli; Ls, lesion in microvilli. Scale bars as indicated. Additional micrographs are provided in **Figure S3**.

The guts of surviving psyllids fed on Cry1Ba1 transgenic Valencia or Duncan or wild type plants for 8 days were dissected and examined by TEM for comparison with those of psyllids fed on transgenic or wild type *B. koenigii* (18). Cry1Ba1-mediated damage to the psyllid gut was noted in all cases for psyllids fed on transgenic plants. In all instances, the midgut microvilli of psyllids fed on Cry1Ba1 plants were swollen or deformed with extensive lesions exposing the gut epithelial cell membrane (**Figure 3**; **Figure S3**). The lesions are associated with thinning of the cytoplasm and cell organelles in the gut epithelium, and disruption or bursting of the cell. Breakdown of the basement membrane overlying the gut epithelium was not observed. These observations are consistent with damage associated with the toxicity of Bt-derived pesticidal proteins, such as Cry1Ba1. The impacts of bacterially expressed Cry1Ba1 on the psyllid gut epithelium, which are similar to those illustrated here are shown in Fernandez-Luna et al. (16).

## 4 Discussion

The primary goal for this research was to assess the impact of transgenic citrus expressing Cry1Ba1 on the survival, physiology and behavior of the Asian citrus psyllid, *D. citri*. This work provides the foundation for the potential use of such transgenic plants as part of a multi-pronged strategy for management of citrus greening. Having confirmed *cry1ba1* gene presence, transcription and translation in different lines of transgenic Indian curry leaf plants (18), Duncan grapefruit, Valencia sweet orange and Carrizo citrange, bioassays were conducted to examine the impact of transgenic Cry1Ba1 plants on ACP. The most dramatic reductions in ACP survival were seen on transgenic Indian curry leaf plants, with significant reductions in psyllid survival also seen on Valencia. Damage to the midgut epithelium typical of Bt pesticidal protein-mediated toxicity was observed, which supports that psyllid mortality was caused by Cry1Ba1 toxicity.

### 4.1 Production of transgenic citrus plants

The expression of Cry1Ba1 in transgenic Valencia and Duncan plants resulted in physiological changes to the plant observed as a “delayed senescence” phenotype. Similar changes in transgenic *B. koenigii* were associated with alterations in plant hormone levels. The abundance of senescence inhibitors increased whereas the abundance of most factors that promote senescence decreased (18). It is unclear whether these physiological changes result from general stress to the plant associated with Cry1Ba1 expression, or from a specific impact of this protein on plant metabolic pathways. The unexpectedly low transformation rates for all of these citrus varieties in comparison to previous reports (23, 34) could also be explained if Cry1Ba1 deleteriously affected the physiology of the regenerating shoots. As noted previously (18), the commercial utility of some bacterial pesticidal proteins has been limited by such impacts on the physiology of the plant.

In contrast to *B. koenigii* where high levels of phenolics interfered with the transfer of soluble proteins to membranes for western blot analysis (**Figure S1**) (18), sufficient soluble plant protein was extracted and transferred for detection of Cry1Ba1 in tissues of transgenic Duncan, Valencia and Carrizo by western blot (**Figure S1**). The predominant immunoreactive band from transgenic plants was ~ 50 kDa, the expected size for the protein for which toxicity has been confirmed by Fernandez-Luna et al. (16) (**Figure S1** **)** (18). In some cases, replicate blots were warranted to confirm expression of Cry1Ba1 of the expected size. However, the immunoreactive band of lower molecular mass detected for some plants could result from degradation of the 50 kDa protein in planta or during processing for western blot (**Figure S1**; **Table S1**). The stability of expressed Cry1Ba1 within the plant and comparison of Cry1Ba1 levels with plant age warrant further investigation.

### 4.2 Impact of plant susceptibility to psyllid feeding on ACP mortality

It is important to note that Cry1Ba1 levels in transgenic plants were not quantified in the current study. Therefore, it is possible that differences in levels of Cry1Ba1 expression between different citrus varieties could account for the differences in psyllid mortality observed in bioassays. In this scenario, Cry1Ba1 expression by the six Carrizo plants used in bioassays, for example, could have been too low for psyllids to acquire a toxic dose within the eight-day bioassay period. (It is notable in this context that Cry1Ba1 detection in the Carrizo plants was more consistent than in the other citrus varieties; **Table S1**).

A second factor that could impact the amount of Cry1Ba1 ingested by the psyllid is the susceptibility of each citrus variety to psyllid feeding. The Indian curry leaf plant is highly attractive to ACP, in large part due to continuous flushing, i.e. the production of new, bright green succulent leaves. Psyllids are highly attracted to such new leaves, which are the favored sites for egg laying and nymph development (35, 36). In contrast, citrus cultivars such as Duncan, Valencia and Carrizo flush intermittently. These differences in plant growth support the use of Indian curry leaf plants as trap plants to intercept psyllids away from fruit-yielding trees, and may also explain the lower psyllid survival in whole plant bioassays on transgenic Indian curry leaf plants compared to that on transgenic Valencia, Duncan or Carrizo plants. As psyllids feed more efficiently on immature leaves (37), psyllids in the *B. koenigii* treatment may have ingested more Cry1Ba1 from the continuous flush than psyllids in other transgenic treatments resulting in the higher mortality levels observed. Conversely, the lack of mortality observed for *D. citri* on transgenic Cry1Ba1 Carrizo plants may have been caused by known tolerance of trifoliate citrus varieties, such as Carrizo, to psyllid feeding as compared with non-trifoliate citrus such as Valencia (38). By using electrical penetration graph recording, Willett et al. (39) demonstrated significantly reduced phloem feeding behaviors by ACP on all *Poncirus* accessions tested, including Carrizo, compared to a wide variety of non-*Poncirus* citrus genotypes, including Valencia. The fibrous ring (sclerenchyma) around the phloem of x*Citroncirus* accessions (hybrids of *Poncirus trifoliata* and *Citrus* spp.) is thicker than that in other citrus genotypes, which is associated with reduced penetration of the vascular bundle and phloem feeding by ACP on *Poncirus* hybrids, such as Carrizo (40). Taken together, the impact of a transgenic citrus variety expressing a bacterial pesticidal protein will be influenced by the susceptibility of that variety to psyllid feeding and the amount of pesticidal protein ingested. Consequently, the use of bacteria-derived pesticidal proteins, or indeed other bioactives for transgenic tolerance to ACP, may be limited to a subset of citrus varieties such as Valencia sweet orange. The potential importance of plant susceptibility to psyllid feeding to psyllid mortality outcomes requires further investigation (41).

In choice test assays, psyllids did not discriminate between transgenic and control plants, which is to be expected. Differences may be observed following onset of feeding however: on acquisition of sufficient pesticidal protein to cause damage to the gut epithelium, insects commonly stop feeding and may die due to starvation. Analysis of feeding behavior using the electrical penetration graph system would shed light on the impact of Cry1Ba1 on psyllid feeding behavior, and potential differences between citrus varieties (36).

### 4.3 Possible applications of transgenic trees in citrus

The successful deployment on a commercial scale of transgenic forest trees expressing one or more bacterial pesticidal proteins (13, 42) provides a precedent for the use of transgenic perennial citrus trees, which differ markedly from traditional Bt crops that are planted annually. Bacterial pesticidal proteins have been combined with insecticidal agents with different modes of action (e.g. protease inhibitors) in these trees for broader suppression of pest species and to delay the onset of insect resistance.

We envision the possibility of applying genetically engineered citrus trees in citrus greening management that extends beyond sole cultivation of fully transgenic crops. For example, the Cry1Ba1-expressing Indian curry leaf plant could be planted adjacent to non-transgenic citrus and serve as a toxic trap plant. A logical next step would be to combine the Bt-expressing trap crop with a psyllid deterrent, such as kaolin (43, 44), applied to fruit-yielding citrus. The concept of combining a pest deterrent (“push”) with an alternative, more attractive host plant modified or engineered to produce an insecticidal agent (“pull”) was introduced in a prescient paper by Miller and Cowles in 1990 (45) and has been suggested as a strategy for management of *D. citri* (46). Furthermore, the efficacy of this tactic has now been confirmed in multiple field studies employing *B. koenigii* given that this host is: 1) more attractive to *D. citri* than *Citrus* genotypes and thus outcompetes the cultivated crop when planted so as to surround the cultivated crop (47–49) and 2) is immune to the pathogen causing citrus greening (50). The use of a transgenic trap plant rather than transgenic fruit-yielding trees may also align more readily with public sentiment regarding bioengineered food products (51).

### 4.4 Considerations for deployment in the field

Insect resistance management is a primary concern for the sustainable use of transgenic crops that express pesticidal proteins such as Cry1Ba1 (52, 53). One important component for successful resistance management has been the deployment of transgenic plants expressing multiple bioactives with different modes of action (e.g. bacterial pesticidal proteins from different structural classes (14); referred to as pyramiding) as the use of combined bioactives delays the onset of insect resistance (54). With such multiple traits targeting the same pest, if resistance develops to one, the target pest would succumb to the toxicity of a second bioactive. Even with multiple traits however, additional resistance management measures are needed for long-term pest management.

Insect resistance has been successfully managed using a high-dose/refuge strategy. In this case plants express a dose sufficient to kill insects that are heterozygous for resistance, and a refuge is used for maintenance of susceptible insect genotypes in the pest population. Following mating of these susceptible insects from the refuge with resistant insects, the heterozygous progeny are unable to survive on the transgenic plants. Key factors pertaining to the success of this approach are sufficient dispersal of the pest insect to facilitate mating between individuals from refuges with those from the transgenic crop, and a fitness cost associated with the resistance phenotype (52). In the case of ACP, while psyllids are capable of moving 1–2 km within a week (55), these long-distance migrations are relatively rare and most likely when resources (i.e. new leaf flush) become limiting (55, 56). Having settled on appropriately flushing host plants, psyllid populations typically move < 15 m (57). Such limited dispersal behavior would not be favorable for resistance management in groves planted entirely to transgenic citrus. This insect behavior would be appropriate however for effective resistance management on deployment of transgenic trap plants. Such trap plants would be planted around the grove or interspersed among non-transgenic fruit-bearing citrus trees. These resistance management considerations provide an additional argument for the use of trap plants for psyllid control in citrus rather than use of transgenic fruit-bearing citrus trees. Taken together, the use of a pyramid of bioactives from trap plants with proximal nontransgenic trees serving as refuge, within an integrated pest management approach may offer the optimal strategy for use of pesticidal proteins in citrus. The fact that ACP has not previously been exposed to bacteria-derived pesticidal proteins to any extent is an additional benefit due to the absence of pre-existing resistance.

### 4.5 Major conclusions and future research

The results from this study support the concept for use of transgenic citrus or trap plants for psyllid management as one component in an integrated and multipronged approach for citrus greening management. The bioassay data for Indian curry leaf plants along with resistance management considerations are particularly compelling for a push-pull, trap plant-based strategy. Several research avenues will allow for refinement of this transgenic plant approach including peptide- or mutagenesis-mediated optimization of Cry1Ba1 toxicity as previously demonstrated against other hemipteran pests (58–61), and screening for bacterial pesticidal proteins with greater natural toxicity to *D. citri* toward ready attainment of a high-dose approach. As pyramiding of bioactives has proved to be a successful approach for the deployment of insect resistant transgenic plants (52), the combined action of multiple pesticidal proteins or pesticidal proteins in combination with other bioactives such as gene silencing RNAs (62) against *D. citri* warrants investigation. Laboratory-based selection of *D. citri* for resistance to Cry1Ba1 and other traits selected for use in a pyramid approach would provide insight into the potential fitness costs associated with resistance should it arise in the field. In the long term, whether transgenic plants are adopted for *D. citri* management in citrus will be determined by industry.

## Data availability statement

The original contributions presented in the study are included in the article/**Supplementary Material**. Further inquiries can be directed to the corresponding author.

## Author contributions

SR, VO, FI-C, LS, DA, TS, CE-M, RB performed the experiments. VO, FI-C, LS, BB designed the experiments. VO and BB wrote the manuscript. All authors contributed to the article and approved the submitted version.
