# Supplementary material for: Cry1Ba1-mediated toxicity of transgenic Bergera koenigii and Citrus sinensis to the Asian citrus psyllid Diaphorina citri
Source: Front Insect Sci. 2023 Apr 24;3:1125987. doi: 10.3389/finsc.2023.1125987 (PMC10926525; doi:10.3389/finsc.2023.1125987)

Supplementary Material

1 Supplementary Figures and Tables

1.1 Supplementary Figures

Supplementary Figure 1. Western blot confirmation of Cry1Ba1 and GFP protein expression. **A.** IgG purified from the polyclonal anti-Cry1Ba1 antiserum was sufficiently sensitive to detect 0.02 µg Cry1Ba1. **B-H:** Western blot analyses for detection of both Cry1Ba1 and GFP in the tissues of transformed plants of Duncan, Valencia, Carrizo, and *Bergera koeniggi* (BER; Ravanfar *et al*., 2022) are shown. Western blots for detection of Cry1Ba1 were conducted before stripping the membrane and reprobing for GFP detection. For all western blots, bands of the targeted protein are boxed. Arrows indicate an immunoreactive Cry1Ba1 degradation product. Table S2 summarizes the Cry1Ba1 detection results from these figures.

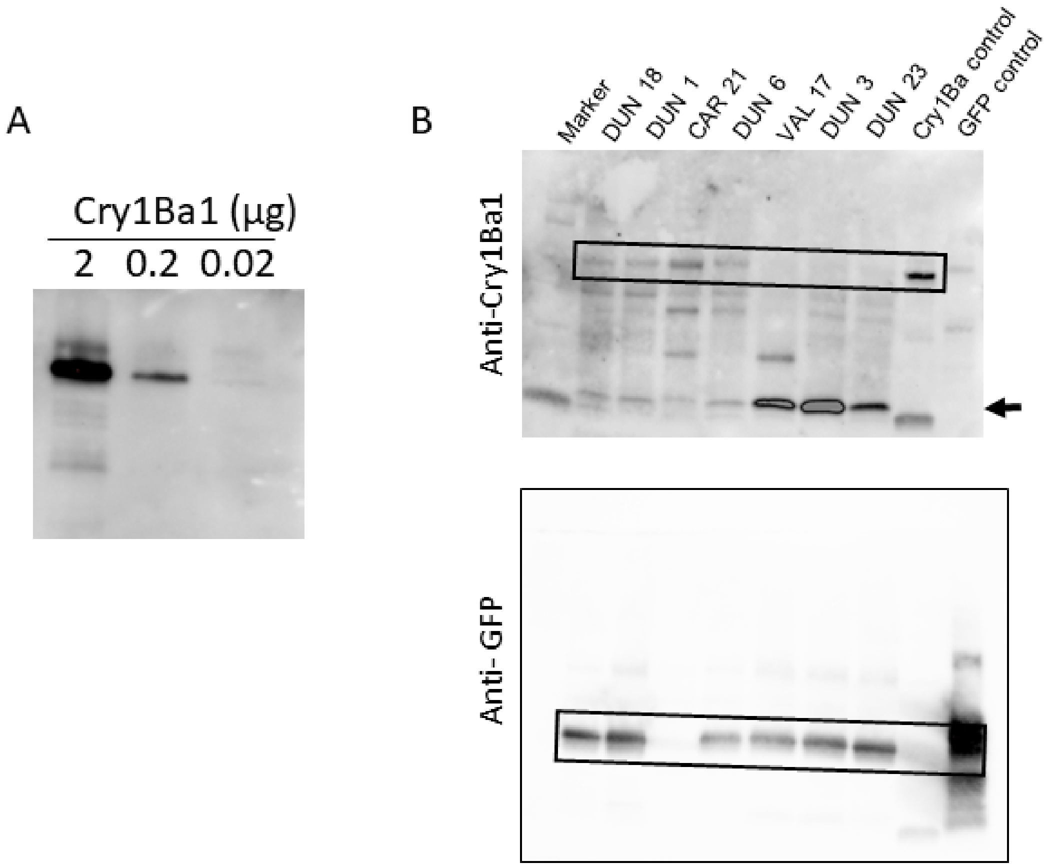

Supplementary Figure 1 ctd.

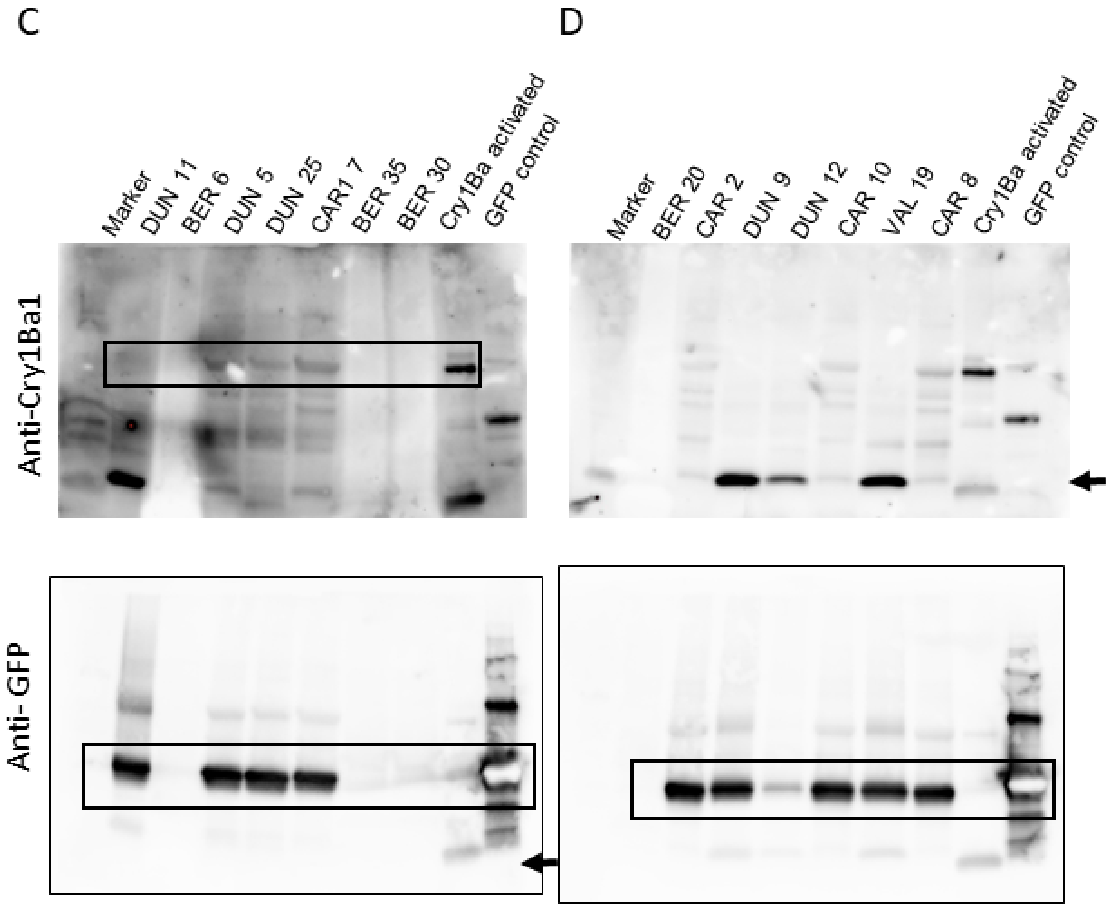

Supplementary Figure 1 ctd.

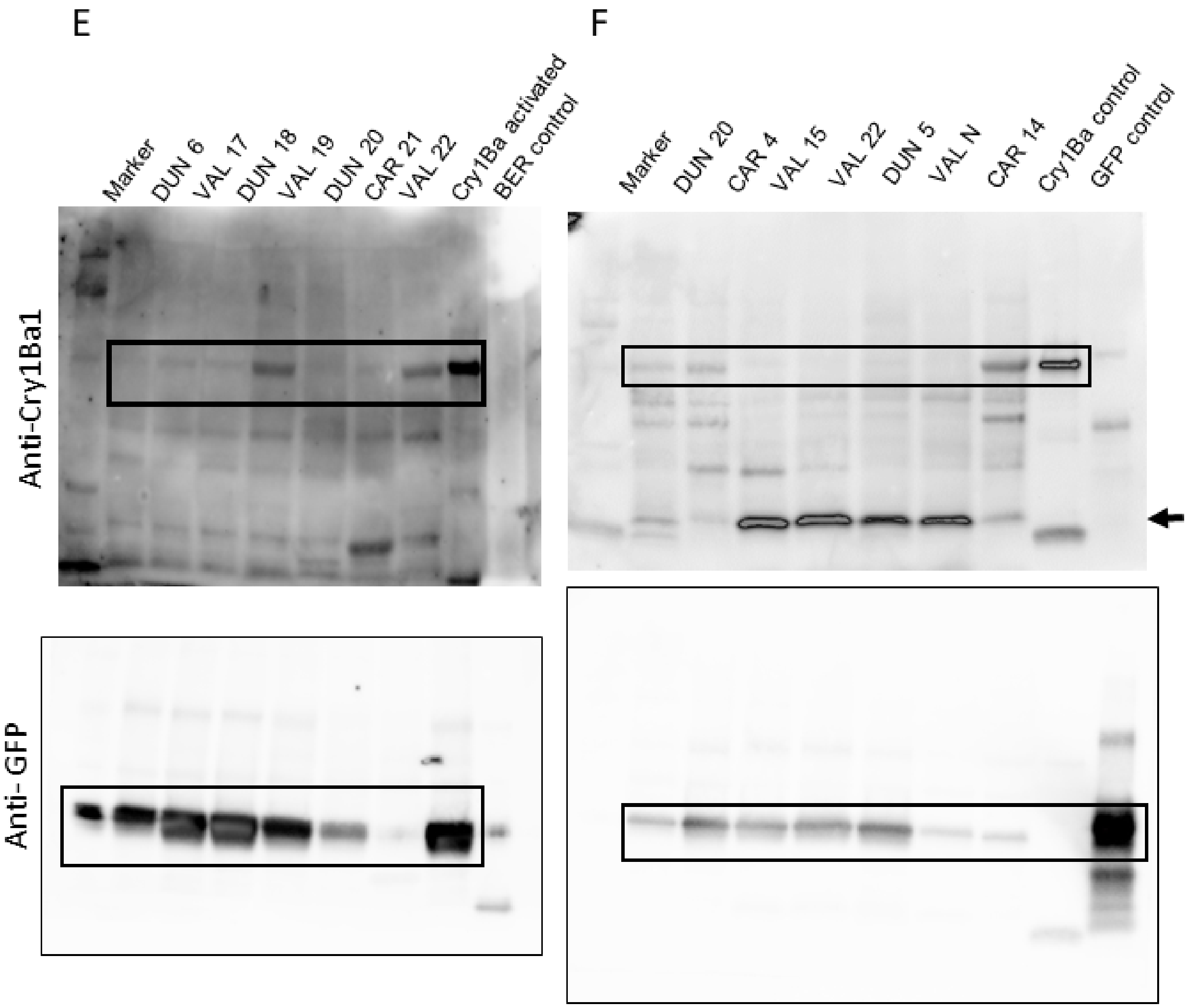

Supplementary Figure 1 ctd.

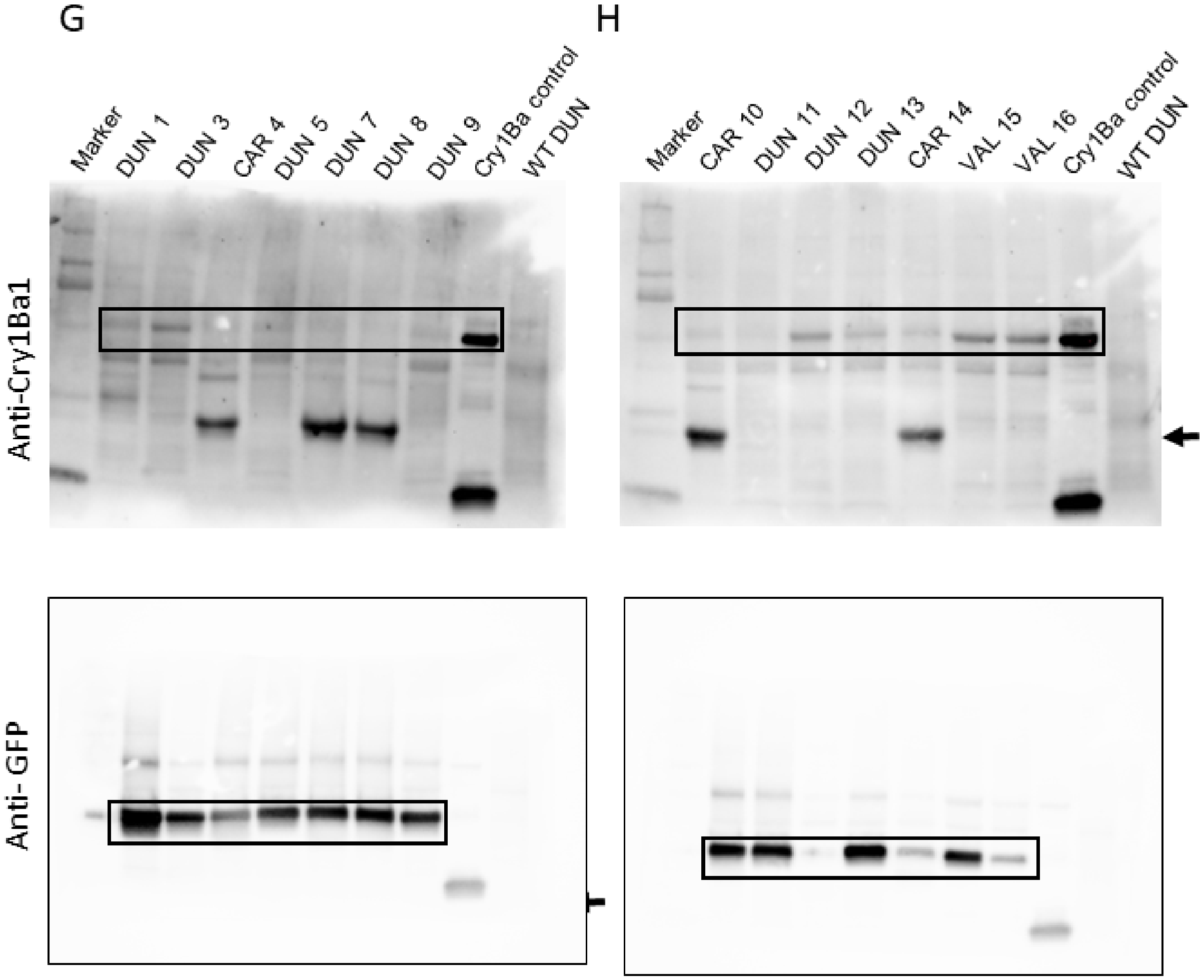

1.2 Supplementary Table

Supplementary Table 1. Summary of western blots to confirm Cry1Ba1 expression in transgenic Duncan, Valencia and Carrizo. The detection of Cry1Ba1 by western blots shown in Fig. S1 panels B- H is indicated. The purpose of these blots was to confirm Cry1Ba1 expression, not for comparison of Cry1Ba1 levels between transgenic lines. +, detected; ++, strong band; -d, protein expressed but degraded based on immunoreactive products of ~20kDa. Note the variation between blots both in terms of detection and band intensity. Proteins of ~50 kDa or immunoreactive bands presumed to be Cry1Ba1 degradation products, were detected for all transgenic plants suggesting that Cry1Ba1 was expressed in all transgenic plants.

Plant	Cry1Ba1 detection shown in Fig. S3 panels B-H							Conclusion
	B	C	D	E	F	G	H	
DUN WT						-	-	-
DUN 1	+					+		+
DUN 3	-d					+		+
DUN 5		+		-d		(+)		+
DUN 6	+			+				+
DUN 7						-d		-
DUN 8						-d		-
DUN 9			-d		+			+
DUN 11		-d					(+)	-
DUN 12			-d				++	+
DUN 13							+	+
DUN 18	+			+				+
DUN 20				(+)	+			+
DUN 23	-d							-
DUN 25		+						+
VAL 15					-d		++	+
VAL 16							++	+
VAL 17	-d			+				+
VAL 19			-d	++				+
VAL 22				++	-d			+
VAL N					-d			-
CAR 2			+					+
CAR 4					+	-d		+
CAR 7		+						+
CAR 8			+					+
CAR 10			+				-d	+
CAR 14					+		-d	+
CAR 21	+			-d				+

Supplementary Figure 2. Altered phenotype of Cry1Ba1 transgenic plants did not impact ACP plant preference. No ACP preference for transgenic or control plants. Host plant choice assays were conducted with transgenic Duncan grapefruit expressing Cry1Ba1, with three independent replicates. There was no significant difference in the numbers of psyllids that selected the transgenic versus control plants.

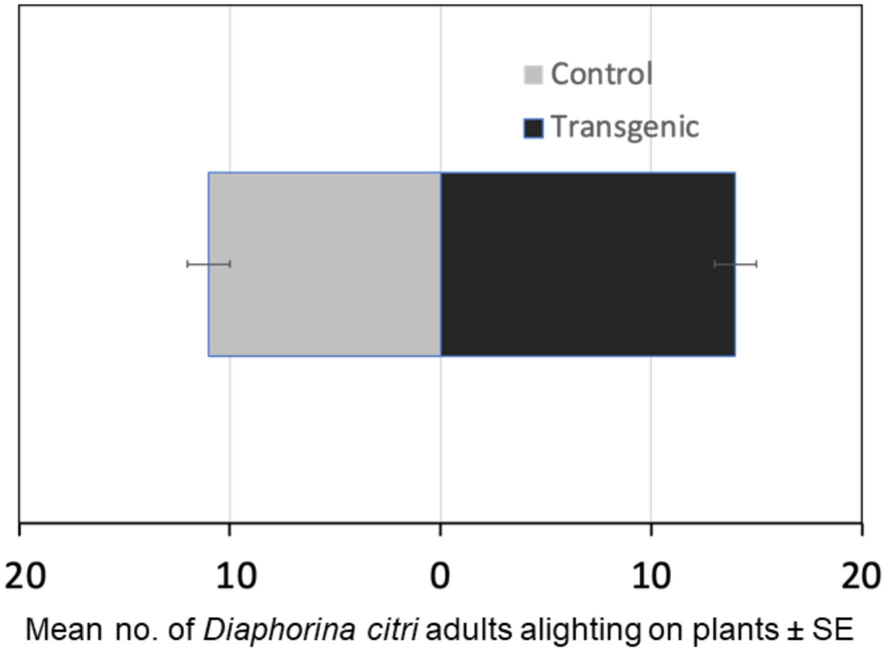

Supplementary Figure 3. Damage to the gut epithelium of *Diaphorina citi* following feeding on Cry1Ba1-expressing transgenic plants. Supplementary transmission electron micrographs showing the gut epithelia of psyllids fed on WT or transgenic Cry1Ba1-expressing plants. L, gut lumen; mv, microvilli; Ls, lesion. Scale bars as indicated.

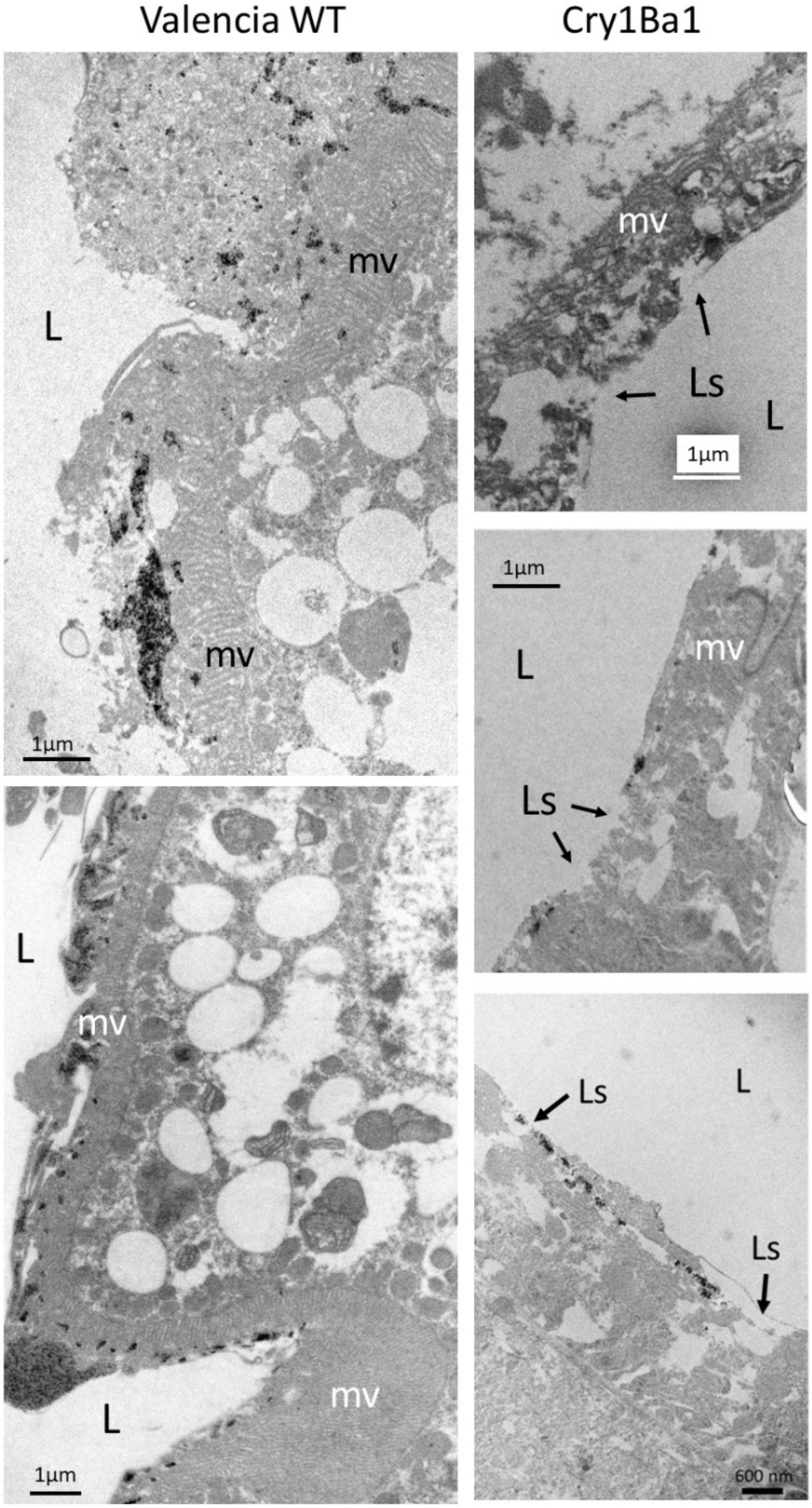

Supplementary Figure 3 ctd.

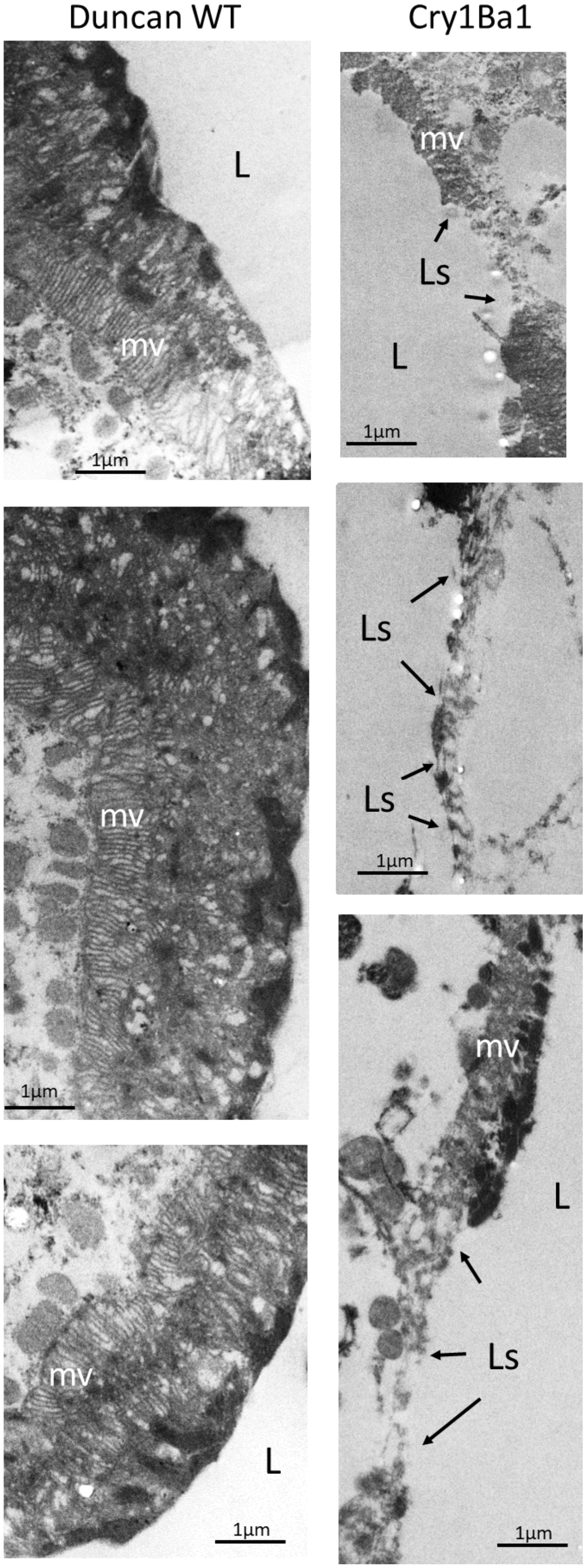

Supplementary Figure 3 ctd.

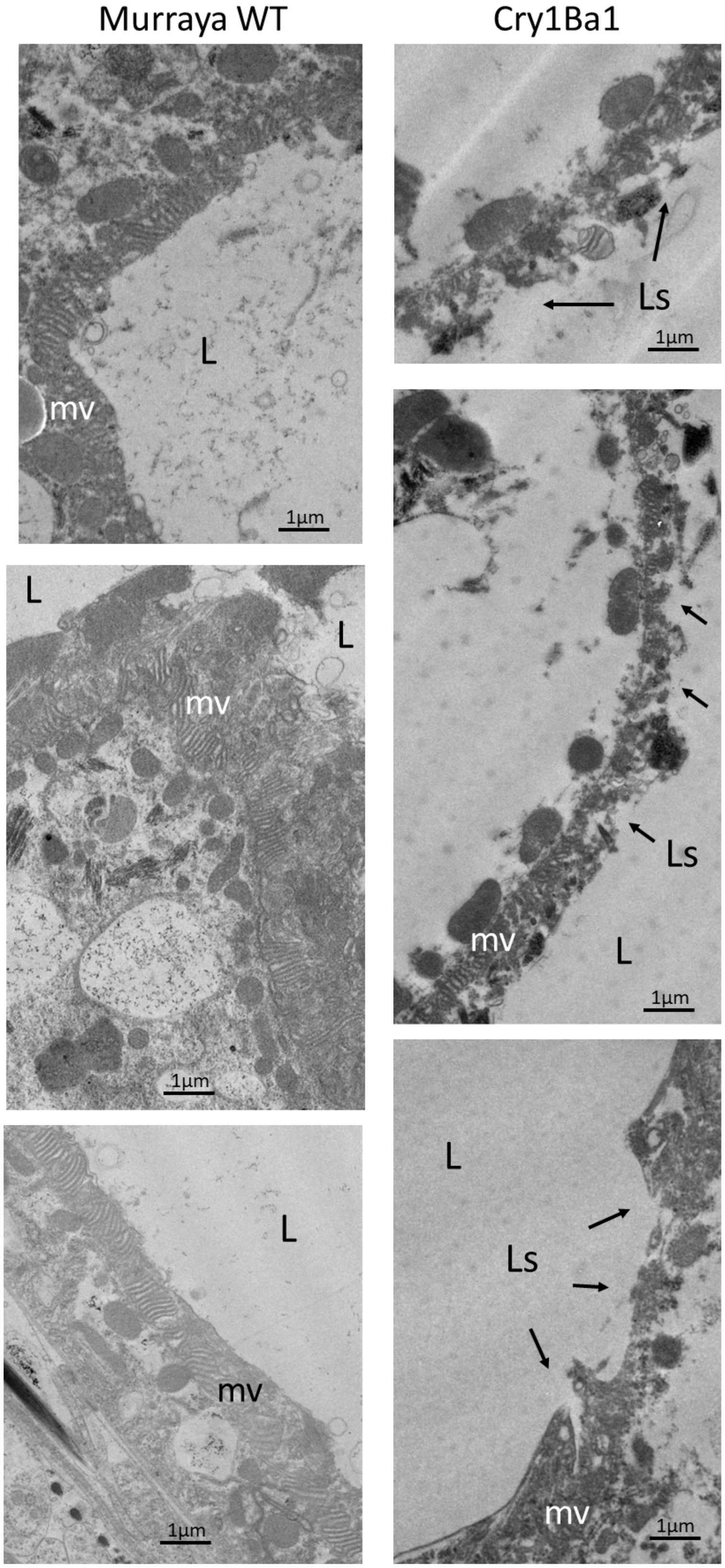

Supplement: Supplementary file 1 [file DataSheet_1.pdf]
